# Histone Acetyltransferase p300 Mediates Histone Acetylation of PS1 and BACE1 in a Cellular Model of Alzheimer's Disease

**DOI:** 10.1371/journal.pone.0103067

**Published:** 2014-07-22

**Authors:** Xi Lu, Yushuang Deng, Daohai Yu, Huiming Cao, Li Wang, Li Liu, Caijia Yu, Yuping Zhang, Xiuming Guo, Gang Yu

**Affiliations:** 1 Department of Neurology, The First Affiliated Hospital of Chongqing Medical University, Chongqing, P.R. China; 2 Laboratory Research Center, The First Affiliated Hospital of Chongqing Medical University, Chongqing, P.R. China; 3 Department of Clinical Sciences, Temple University School of Medicine, Philadelphia, Pennsylvania, United States of America; 4 Department of Bio-therapy & Hemato-oncology, Chingqing Cancer Institute, Chongqing, P.R. China; 5 Department of Neurology, Chongqing Hospital of Traditional Chinese Medicine, Chongqing, P.R. China; 6 Genetics and Molecular Biology, Northwestern University, Evanston, Illinois, United States of America; University of S. Florida College of Medicine, United States of America

## Abstract

Epigenetic modifications, particularly histone acetylation, have been implicated in Alzheimer's disease (AD). While previous studies have suggested that histone hypoacetylation may regulate the expression of genes associated with memory and learning in AD, little is known about histone regulation of AD-related genes such as Presenilin 1(PS1) and beta-site amyloid precursor protein cleaving enzyme 1(BACE1). By utilizing neuroblastoma N2a cells transfected with Swedish mutated human amyloid precursor protein (APP) (N2a/APPswe) and wild-type APP (N2a/APPwt) as cellular models of AD, we examined the alterations of histone acetylation at the promoter regions of PS1 and BACE1 in these cells. Our results revealed that histone H3 acetylation in PS1 and BACE1 promoters is markedly increased in N2a/APPswe cells when compared to N2a/APPwt cells and control cells (vector-transfected), respectively, causing the elevated expression of PS1 and BACE1. In addition, expression of histone acetyltransferase (HAT) adenoviral E1A-associated 300-kDa protein (p300) is dramatically enhanced in N2a/APPswe cells compared to N2a/APPwt and control cells. We have further demonstrated the direct binding of p300 protein to the PS1 and BACE1 promoters in N2a/APPswe cells. The expression levels of H3 acetylation of the PS1 and BACE1 promoters and p300 protein, however, were found to be not significantly different in N2a/APPwt cells when compared to controls in our studies. Furthermore, curcumin, a natural selective inhibitor of p300 in HATs, significantly suppressed the expression of PS1 and BACE1 through inhibition of H3 acetylation in their promoter regions in N2a/APPswe cells. These findings indicated that histone acetyltransferase p300 plays a critical role in controlling the expression of AD-related genes through regulating the acetylation of their promoter regions, suggesting that p300 may represent a novel potential therapeutic target for AD.

## Introduction

Alzheimer's disease (AD) is a progressive neurodegenerative disorder [1]. Numerous studies have indicated that epigenetic modification is involved in the etiology of AD [2]. Of the epigenetic mechanisms, histone acetylation has been unambiguously implicated in the characteristic symptoms of AD such as cognitive dysfunctions [3]–[5]. By neutralizing the electrostatic affinity of DNA and proximal histone, histone acetylation promotes DNA unwinding and makes its chromatin structures accessible to DNA transcription factors, leading to potential transcriptional activations. The process of histone acetylation is mediated by histone acetyltransferases (HATs) and histone deacetylases (HDACs), and a controlled balance of acetylation-deacetylation is essential for cellular homeostasis [6].

To the best of our knowledge, the role of histone acetylation is divergent among several AD *in vitro* and *in vivo* models. Several studies have reported histone hypoacetylation in genes related to memory and synapsis plasticity, indicating that decreased histone acetylation is associated with AD [3], [4]. However, other groups found that histone hyperacetylation is causally linking to AD [7]–[9]. Marambaud *et al*. found that PS1 mutations related to familial AD repress proteasomal degradation of HAT CREB-binding protein (CBP) and upregulate CREB-mediated gene transcription in murine neuronal cells [7]. These inconsistent results indicated that the levels of histone acetylation vary depending on brain regions, animal models, cell types and gene loci [10]. Currently, little direct evidence has been presented to link AD-related genes to histone modification.

Among the nuclear HATs, the intrinsic E1A-associated 300-kDa protein (p300), sharing high conserved regions with CBP, plays an important role in familial AD development. PS1 mutations promote expression of CBP/p300 in cultured neurons and inactivated PS1/2 reduces the expression of CBP/p300 in conditional knockout mice [7], [8]. In addition, Gu *et al*. reported an enhancement of CBP/p300 in human neuroblastoma SH-SY5Y cells by Aβ peptide overproduction [11], [12]. CBP/p300 is also involved in signaling mechanisms underlying neuronal apoptosis and long-term memory deficiencies. Rouaux *et al*. pointed out that fine-tuning of CBP/p300 plays a neuroprotective function through their HAT activity domain and the loss or overexpression of CBP/p300 is responsible for the neuronal death [13]. Oliveira *et al*. have reported that deficiency of acetyltransferase CBP/p300 loss is associated with memory deficits and synapsis dysfunction [14]. So far there is no evidence on whether p300 is involved in the regulation of transcription of the AD-related genes.

In this study, we have demonstrated for the first time the alterations of histone acetylation H3 in the PS1 and BACE1 promoter regions by utilizing an *in vitro* model of a murine neuroblastoma cell line in which Aβ deposits are induced by transfection with Swedish human APP695 (N2a/APPswe) [15], and we have also established the critical role of p300 in the regulation of histone acetylation in PS1 and BACE1 by using curcumin, a natural selective inhibitor of p300 in HATs [16], [17]. Thus, we have provided supporting evidence that histone acetylation of AD-related genes is a novel mechanism for familial AD pathology.

## Materials and Methods

### Cell Culture

The murine brain-derived neuroblastoma (N2a) cell lines[1] were performed previously [18]. N2a cell line stably transfected with Swedish human APP695 (APPswe) and wild-type human APP695 (APPwt), respectively, were kindly gifted to us by Dr. Huaxi Xu (Burnham Institute for Medical Research, La Jolla, USA). N2a cells transfected with APPswe, APPwt and empty plasmid vectors (vector-transfected) as controls were cultured in a 1∶1 mixture medium of DMEM and OPTI-MEM supplemented with 5% fetal bovine serum and 150 µg/mL G418.

### Drug Treatment

Curcumin (Sigma, USA) was dissolved in DMSO and added into the medium of APPswe-transfected N2a cells at 10 µM for 48 hours. The cells and cell media were collected separately. Cells were lysed for RNA, protein and chromatin extraction or kinase activity assays as outlined below.

### Total RNA Extraction and Quantitative RT-PCR

Total RNAs were extracted from the cultured cells using Trizol reagent (TaKaRa, Japan) according to the manufacturer's protocol. Following template cDNAs were obtained by reverse transcription (RT) using PrimeScript reverse transcription kit (TaKaRa, Japan), real time PCR was performed empolying Thermal Cycler Dice Real Time System (Thermo, USA) utilizing a one-step SYBR PrimeScript PCR kit (TaKaRa, Japan). The transcripts in cDNAs samples were detected by PCR reactions with primer as follows: Ps1 forward, 5-′GCCAGAATGACAGCCAAGAAC-3′, reverse, 5-′GCTCTTCGTCTTCCTCCTCATC-3′; Bace1 forward, 5-′ GACCTCCGAAAGGGTGTGTAT-3′, reverse, 5-′ AGTCAAAGAAGGGCTCCAAAG-3′; GAPDH forward, 5-′GGTGAAGGTCGGTGTGAACG-3′, reverse, 5-′CTCGCTCCTGGAAGATGGTG-3′ (Sangon Biotech, China). The quantity in each sample was normalized to GAPDH.

### Western Blotting

After N2a/APPswe cells were treated with or without curcumin, total proteins and nuclear proteins from cells were extracted respectively. An equal amount of total proteins and nuclear proteins was separated on polyacrylamide gels and then transferred onto polyvinylidene difluoride (PVDF) membrane (Millipore, USA). After having been blocked with 5% bovine serum albumin (BSA) for 2 hours, membranes were incubated overnight at 4°C probed with primary antibodies: full length APP at 1∶1000 (Epitomic, USA), PS1 at 1∶500 (Epitomic, USA) and BACE1 at 1∶1000 (Epitomic, USA) for total proteins; H3-Ace at 1∶1000 (Millipore, USA) and p300 at 1∶200 (Abcam, USA) for nuclear proteins. After being washed, membranes were then incubated with HRP conjugated secondary antibodies. The immunoreactive protein bands on the membrane were detected with a Chemiluminescence Luminal reagent (Keygen, China), and the densities of the bands were analyzed with Quantity One (Bio-Rad, USA).

### Measurement of Aβ by ELISA

The levels of extracellular amyloid-β peptide Aβ1-40 and Aβ1-42 in media from N2a APPswe, APPwt and control cells were measured, respectively, by sandwich ELISA assay. Experiments were performed in 96-well plates using commercially available kits (BioVision, USA) according to the manufacturer's instructions. Absorbance values at 450 nm were determined.

### HATs Activity Assay

The cultured cells were collected and nuclear proteins were extracted. Nuclear extraction (50 µg) from each group was taken for measurement of HATs activity by using a HATs assay kit (BioVision, USA) according to the manufacturer's instructions, and the absorbance was taken at 450 nm.

### Immunofluorescent Staining

N2a APPswe, APPwt and control cells were fixed with 4% paraformaldehyde for 30 minutes and incubated with 0.4% Triton for 10 minutes at room temperature. The cells were then incubated with p300 antibodies at 1∶50 (Abcam, USA) overnight at 4°C. Cells were washed and incubated with Cy3 conjugated secondary antibody for 60 minutes at 37°C. At last, cells were examined at 400-fold magnifications with a laser scanning confocal microscope.

### Chromatin Immunoprecipitation Assay

A *ChIP* assay was performed using a *ChIP* assay kit (Millipore, USA) according to the manufacturer's protocol. Briefly, after being treated with or without curcumin, N2a/APPswe cells were cross-linked with formaldehyde in culture medium, followed by glycine stop-fix solution. Cells were then collected and chromatins of the cells were cut into small fragments ranging from 200 bp to 800 bp by sonication with the optimized condition (30 pulses per 10 secconds each with a 30 -second rest between pulses and repeated 70 times). The protein–DNA complex was precipitated by acetylation-H3 (Ac-H3) antibody (*ChIP* grade, Abcam, USA) and p300 antibody (*ChIP* grade, Millipore, USA) overnight at 4°C, and non-specific rabbit IgG was precipitated as a control. Following chromatin-antibody complexes were eluted, the cross-links were reversed, and DNA extracted from the immunoprecipitated complex was analyzed by quantitative PCR and Semi-quantitative PCR using primers targeting promoters: PS1 forward, 5-′ TTTGACCCAGTGTATCTTAGTTTG-3′, reverse, 5-′ TCGGGATGGTGTTGTATCTG-3′ (−981 to −862); BACE1 forward, 5-′ AAGACAAAGGGATTCAGGCATAG-3′, reverse, 5-′ AGAGGCTGGAGGAGCAAGGT-3′ (−750 to −592); (Sangon Biotech, China). Each value was normalized to the input DNA of the sample.

### Statistical Analysis

All data were presented as mean ± SD and statistical analyses were carried out using ANOVA with post-hoc tests for subgroup comparisons and/or *T*-tests. A p- value <0.05 was considered statistically significant. The quantitative data of each variable for each group were obtained from 3 to 5 independent experiments performed in duplicate.

## Results

### N2a/APPswe and N2a/APPwt Cells Display an Elevated Expression of APP, PS1, BACE1 and Aβ

**Figure 1:**
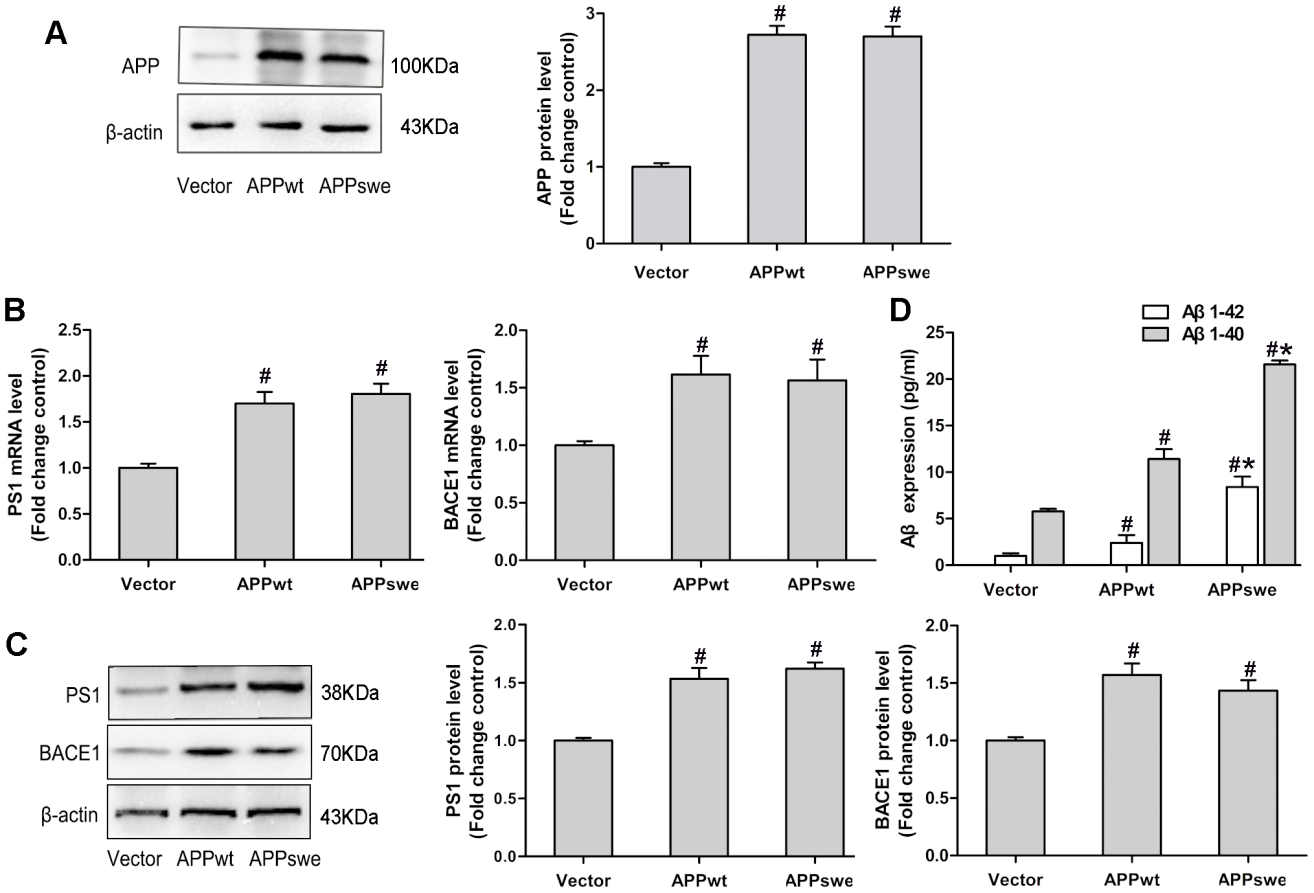
The expression levels of APP, PS1, BACE1 and Aβ were elevated in N2a/APPswe and N2a/APPwt cells. (A) The expression of full length APP was increased in N2a/APPswe and N2a/APPwt cells compared to controls (vector-transfected). (B, C) mRNA and protein levels of PS1 and BACE1 were significantly elevated in N2a/APPswe and N2a/APPwt cells compared with control cells; (D) the downstream expression levels of Aβ1-42 and Aβ1-40 were significantly higher in N2a/APPswe and N2a/APPwt cells than control cells. *: P<0.05, as compared with N2a/APPwt cells, #: P<0.05, as compared with control cells (n = 3).

Mouse neuroblastoma N2a cells stably expressing Swedish mutant APP (APPswe) and wild-type APP (APPwt) have been widely used to study the pathology of AD [15]. We first examined the expression of full length APP in N2a/APPswe and N2a/APPwt cells by the Western blotting. Compared with control cells (vector-transfected), the level of full length APP was significantly higher in N2a/APPswe and N2a/APPwt cells (Figure 1A).We then analyzed the level of PS1 and BACE1 as well as their downstream factors Aβ1-42 and Aβ1-40 in N2a/APPswe and N2a/APPwt cells. As shown in Figure 1B and 1C, both mRNA and protein levels of PS1 and BACE1 were significantly elevated in N2a/APPswe and N2a/APPwt cells compared to control cells Moreover, the expression levels of Aβ1-42 and Aβ1-40 were significantly higher in N2a/APPswe and N2a/APPwt cells than in control cells (Figure 1D). These data indicated that N2a/APPswe and N2a/APPwt cells exhibit increased expression of PS1 and BACE1 and elevated expression of Aβ1-42 and Aβ1-40, characteristics of AD.

### Histone of PS1 and BACE1 Gene Promoters and Cellular Histone Are Hyperacetylated in N2a/APPswe Cells but Not in N2a/APPwt Cells

**Figure 2:**
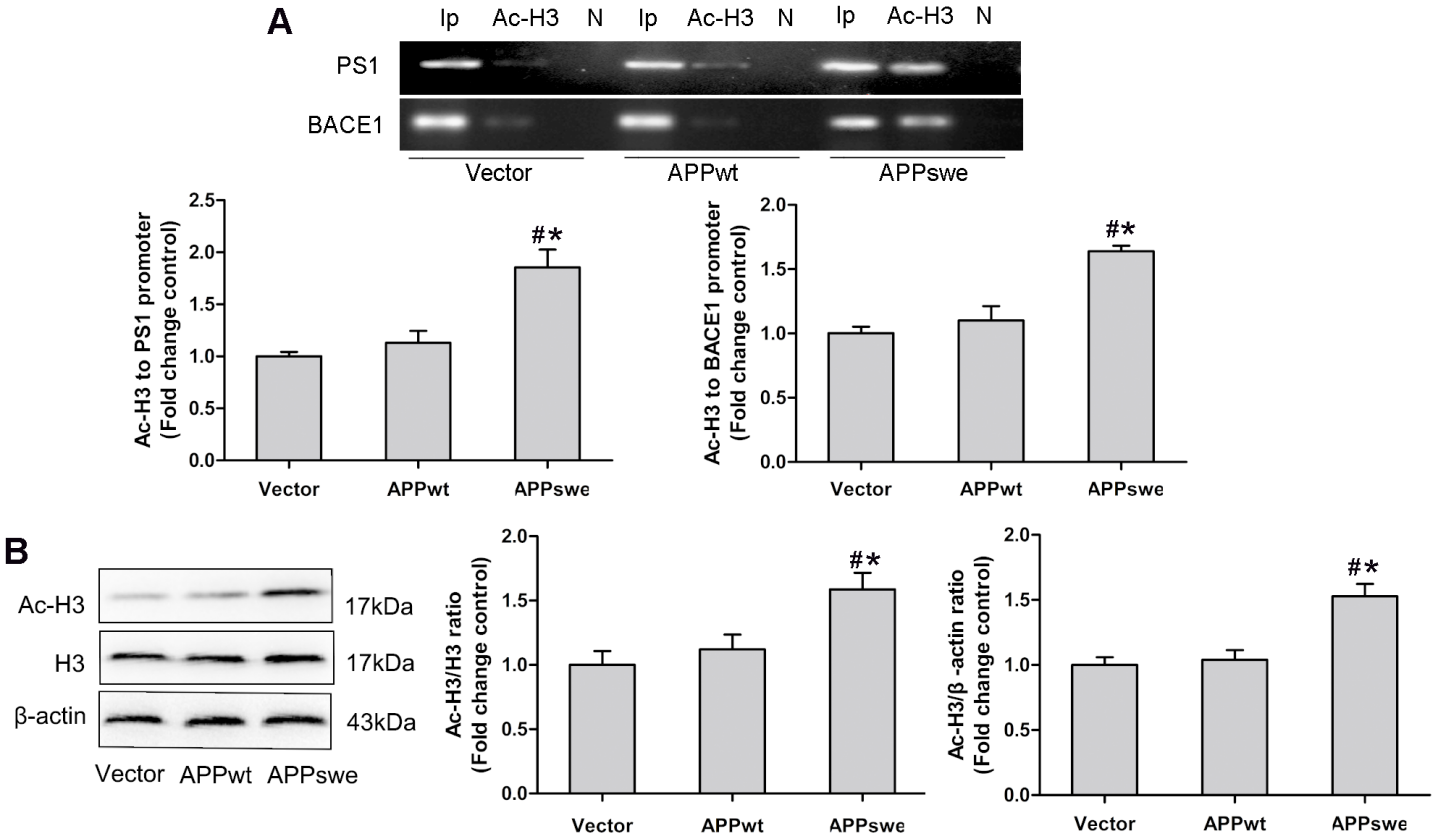
H3 in PS1 and BACE1 promoters and cellular H3 were hyperacetylated in N2a/APPswe cells. (A) the binding of acetylation H3 (Ac-H3) to PS1 and BACE1 promoter regions was markedly higher in N2a/APPswe cells than N2a/APPwt and control cells; (B) the cellular Ac-H3 was increased in N2a/APPswe cells compared with N2a/APPwt and control cells. Ip represents amplification of input DNA from cells; Ac-H3 represents DNA bound to Ac-H3 in the sample; N represents DNA precipitated by normal mouse IgG as negative control. *: P<0.05, as compared with N2a/APPwt cells, #: P<0.05, as compared with control cells (n = 3).

It is well establisehed that histone hyperacetylation is involved in gene activation [6]. Having observed the increased expression of PS1 and BACE1 in N2a/APPswe and N2a/APPwt cells, we therefore decided to examine whether such upregulation of PS1 and BACE1 is due to histone hyperacetylation. For this purpose, we performed a *ChIP* assay to analyze PS1 and BACE1 promoters in N2a/APPswe and N2a/APPwt cells using the acetylation-H3 (Ac-H3) antibody. As shown in Figure 2A, the binding of Ac-H3 to PS1 and BACE1 promoter regions was markedly increased in N2a/APPswe cells but not in N2a/APPwt cells when compared to the control cells. These data suggested that expressions of PS1 and BACE1 are enhanced in N2a/APPswe cells through histone hyperacetylation, likely due to the APPswe mutation, not overexpression of APP in N2a cells. In addition, we examined the whole H3 acetylation by the Western blot in N2a/APPswe and N2a/APPwt cells. As shown in Figure 2B, the cellular H3 acetylation was significantly higher in N2a/APPswe cells than that in control cells. We found, however, only a slightly increasing trend in cellular histone H3 acetylation in N2a/APPwt cells compared to control cells without reaching the statistical significance. The data suggested that the APPswe mutation rather than overexpression of APP in N2a cells induces histone H3 hyperacetylation of PS1 and BACE1.

### Endogenous p300 Was Elevated in N2a/APPswe Cells but Not in N2a/APPwt Cells

**Figure 3:**
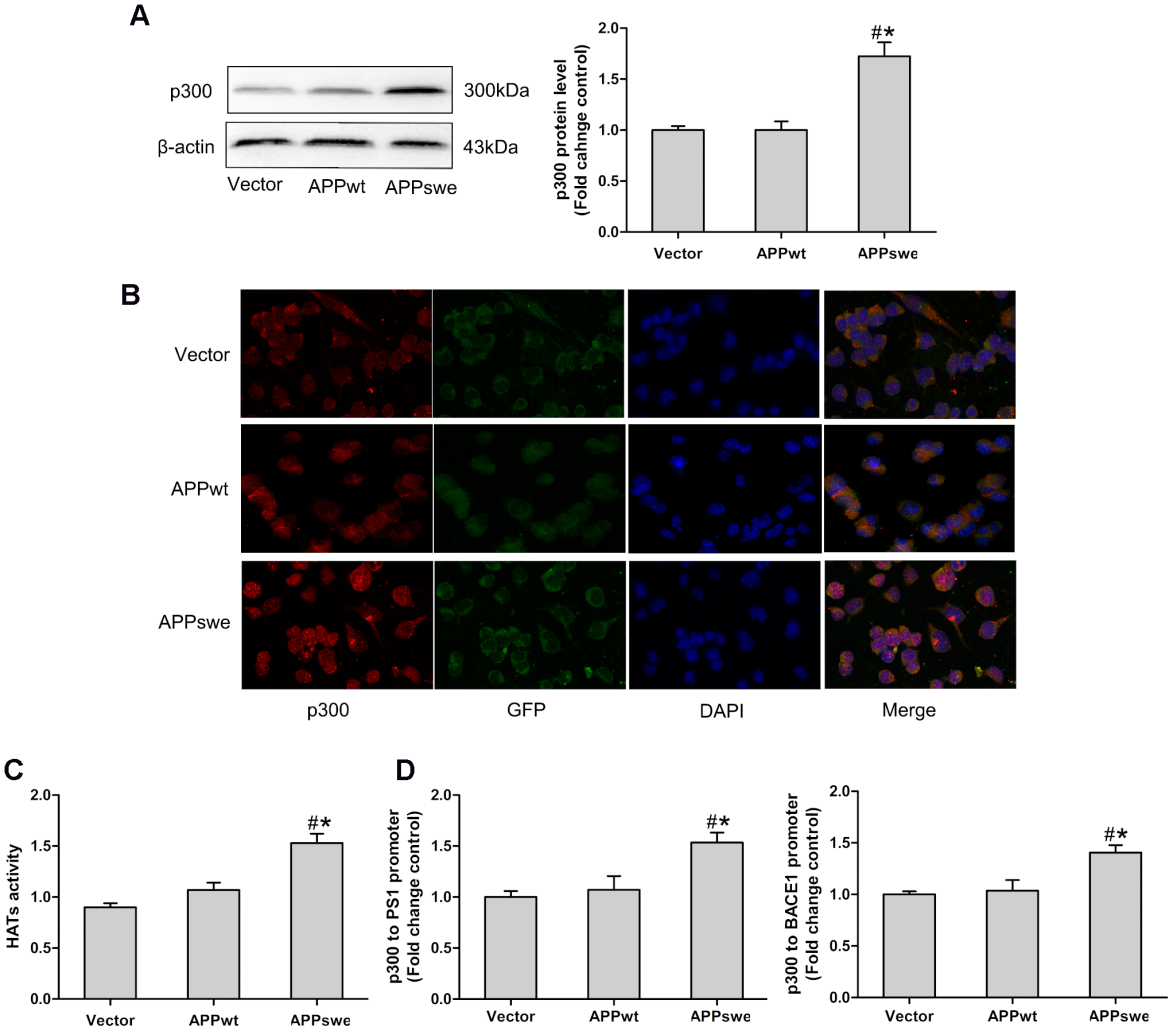
Endogenous p300 was enhanced in N2a/APPswe cells. (A) compared to N2a/APPwt and control cells, the p300 expression was dramatically enhanced in N2a/APPswe cells; (B) overexpression of p300 protein in N2a/APPswe cells was further confirmed by immunocytochemistry, Magnification 400-fold, Scale bar: 50 µm. (C) the HATs activity was significantly higher in N2a/APPswe cells compared to N2a/APPwt and control cells; (D) the level of p300 was higher in the promoter regions of PS1 and BACE1 in N2a/APPswe cells but not in N2a/APPwt cells compared with the controls. *: P<0.05, as compared with N2a/APPwt cells, #: P<0.05, as compared with control cells (n = 3).

The involvement of histone acetylation in the upregulation of PS1 and BACE1 led us to further examine whether p300/CBP, a ubiquitous histone acetyltransferase, dictates the histone acetylation of PS1 and BACE1 in N2a/APPswe cells. We first examined the endogenous p300 expression by Western blot in N2a/APPswe and N2a/APPwt cells. As shown in Figure 3A, compared to control cells, the p300 protein expression was dramatically enhanced in N2a/APPswe cells but not in N2a/APPwt cells. Elevated p300 protein in N2a/APPswe cells was further confirmed by immunocytochemistry (Figure 3B). Furthermore, we examined by ELISA the HATs activity that is involved in the alteration of histone acetylation and found it to be significantly increased in the N2a/APPswe cells but not in the N2a/APPwt cells compared to the controls (Figure 3C). The increased endogenous p300 and HATs activity were consistent with the enhanced Ac-H3 in the PS1 and BACE1 promoters in N2a/APPswe cells (Figure 2A). The association between increased p300 protein and hyperacetylation of PS1 and BACE1 implied that HAT p300 may regulate PS1 and BACE1 expression in N2a/APPswe cells through modulation of histone acetylation in the gene promoters.

### p300 Directly Bound at PS1 and BACE1 Promoters in N2a/APPswe Cells but Not in N2a/APPwt Cells

p300 has been demonstrated to regulate gene transcription via physically binding with their promoters [19]. To gain insight into the molecular basis of epigenetic regulation in the expression of PS1 and BACE1, we next examined whether p300 can physically interact with the promoters in N2a/APPswe cells. We performed *ChIP* assays using promoter specific primers on the PS1 and BACE1 genes with anti-p300 antibody. As shown in Figure 3D, a significantly higher level of p300 was detected in the promoter regions of PS1 and BACE1 in N2a/APPswe cells but not in N2a/APPwt cells compared with the controls. The data suggested that p300 regulate PS1 and BACE1 expression in N2a/APPswe cells by directly binding with the promoters of these genes. It implied that APPswe mutation rather than overexpression of APP induce the p300 protein that is accessible for the promoters of these genes.

### Hyperacetylation of PS1 and BACE1 Was Suppressed by p300 Specific Inhibitor Curcumin in N2a/APPswe Cells

**Figure 4:**
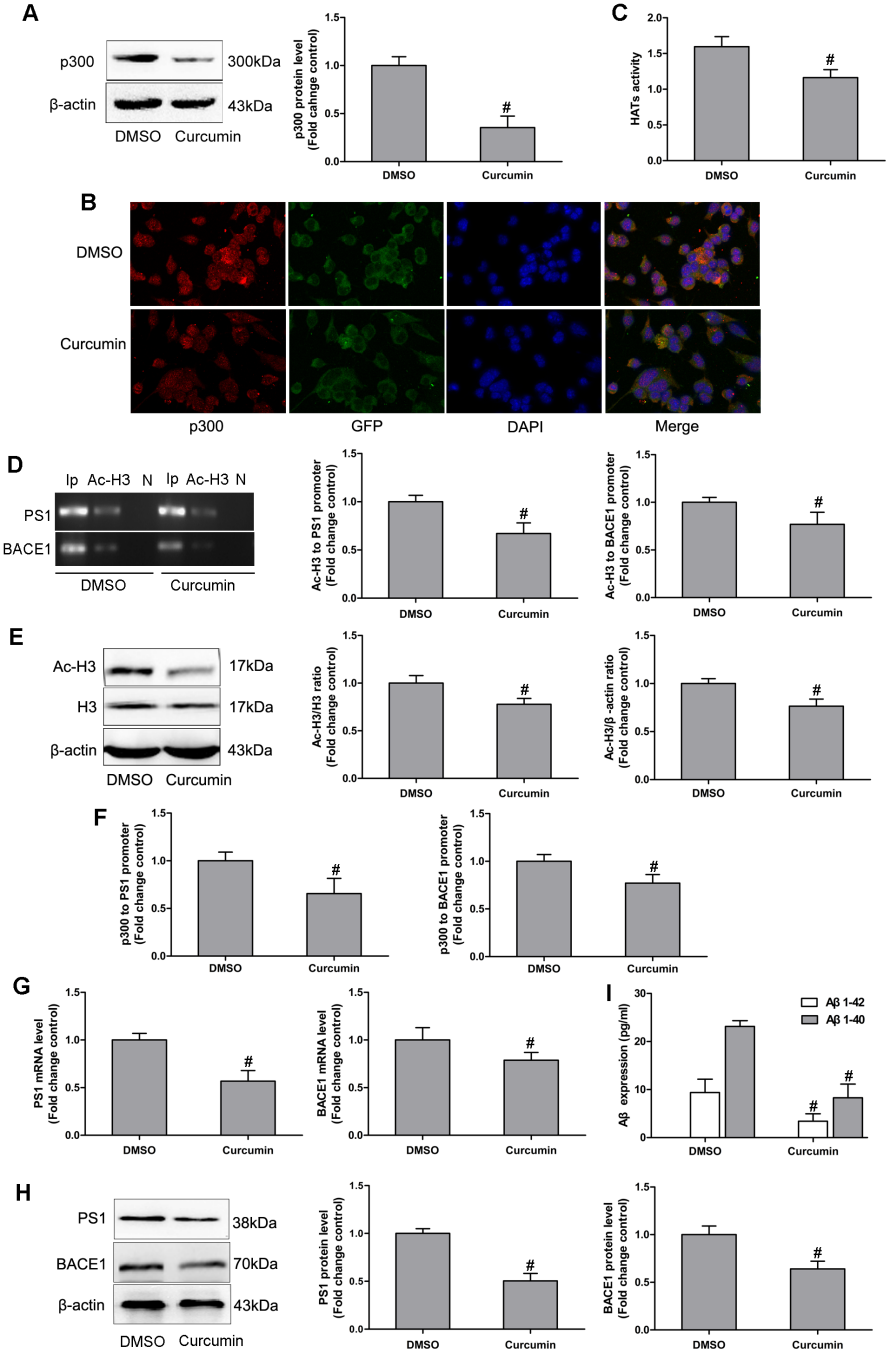
Hyperacetylation of PS1 and BACE1 was suppressed by p300 specific inhibitor curcumin in N2a/APPswe cells. (A) the p300 protein was markedly decreased in N2a/APPswe cells after being treated with curcumin; (B) curcumin dramatically reduced the enhancement effect of p300 in N2a/APPswe cells by immunocytochemistry, Magnification 400-fold, Scale bar: 50 µm; (C) curcumin treatment significantly suppressed the HATs activity in N2a/APPswe cells; (D) the Ac-H3 to PS1 and BACE1 promoter regions was much lower in N2a/APPswe cells after being treated with curcumin compared to DMSO (placebo) group; (E) the cellular Ac-H3 in N2a/APPswe cells was statistically significantly decreased after curcumin treatment; (F) N2a/APPswe cells after being treated with curcumin showed reduced binding of p300 to PS1 and BACE1 in N2a/APPswe cells. (G, H, I) the PS1 and BACE1 expression, followed by their downstream products Aβ1-42 and Aβ1-40, were markedly reduced in N2a/APPswe cells after curcumin treatment. Ip represents amplification of input DNA from cells; Ac-H3 represents DNA bound to Ac-H3 in the sample; N represents DNA precipitated by normal mouse IgG as negative control. #: P<0.05, as compared with DMSO treatment cells (n = 3).

Curcumin, a natural polyphenol of *Curcuma longa*, is shown to be a selective inhibitor of p300 in HATs, via blocking HAT activity and histone acetylation [16], [17]. To test whether the expression of PS1 and BACE1 relies on p300, we examined the histone acetylation of PS1 and BACE1 in N2a/APPswe cells after curcumin treatment. N2a/APPswe cells were cultured with 20 µM curcumin for 48 hours. We first tested whether the curcumin treatment inhibits the p300 expression in N2a/APPswe cells by Western blot and immuocytochemistry. As shown in Figure 4A and 4B, the p300 protein was markedly decreased in N2a/APPswe cells after curcumin treatment, confirming the inhibitory effect of curcumin on p300. Furthermore, curcumin treatment significantly suppressed the HATs activity in N2a/APPswe cells (Figure 4C). These data confirmed that curcumin antagonizes p300, which consequently inhibits the HATs activity.

To explore whether p300 mediates PS1 and BACE1 acetylation, we examined the alteration of Ac-H3 in the PS1 and BACE1 promoters after curcumin treatment by *ChIP* assay. As shown in Figure 4D, the Ac-H3 to PS1 and BACE1 promoter regions were decreased in N2a/APPswe cells after curcumin treatment compared to DMSO (i.e. no curcumin treatment). These data indicated that endogenous p300 plays a role in regulating PS1 and BACE1 hyperacetylation in N2a/APPswe cells. We also examined the cellular Ac-H3 change in N2a/APPswe cells after curcumin treatment by Western blot. The downward trend of cellular Ac-H3 in N2a/APPswe cells was statistically significant with curcumin treatment (Figure 4E).

To explore the functions of HATs activity in the p300 protein, we examined the physical interaction of p300 with PS1 and BACE1 after curcumin treatment. N2a/APPswe cells after curcumin treatment exhibited reduced binding of p300 to PS1 and BACE1 (Figure 4F). These data suggested that the binding of p300 to these genes is decreased due to inhibition of HATs activity within the gene promoters.

Since the hyperacetylation of PS1 and BACE1 was responsible for their active transcription, we further tested whether curcumin treatment could alter the expression of PS1 and BACE1 in N2a/APPswe cells. As shown in Figure 4G and 4H, the PS1 and BACE1 expression levels, followed by their downstream factors Aβ1-42 and Aβ1-40 (Figure 4I), were dramatically reduced in N2a/APPswe cells after curcumin treatment compared with DMSO group. These data confirmed the critical role of p300 in mediating the PS1 and BACE1 expression.

## Discussion

The pathogenesis of AD has not been fully understood yet. Recently, several studies have reported that histone acetylation, one of the epigenetic modifications, contributes to AD development [3]–[5], [20]. AD is characterized by a major feature of Aβ plaques that undergo sequential cleavages of amyloid precursor protein (APP) by β- secretase (BACE) and γ-secretase at the N- and C-termini ends of Aβ sequence, respectively [1]. Indeed, the expression level of PS1, the catalytic core component to γ-secretase, as well as the expression and activity of BACE1 are elevated in the brains of AD patients and animals [21]–[23]. Mechanisms related to epigenetic regulation of AD-associated genes such as PS1 and BACE1 are still inconclusive. In the present study, we utilized N2a cells that were transfected with Swedish human APPswe (N2a/APPswe) and wild-type APP (N2a/APPwt) as cellular models for AD [15]. We first verified the higher expression of APP in N2a/APPswe and N2a/APPwt cells than that of control cells. Then we found that mRNA and protein levels of PS1 and BACE1 were significantly elevated, followed by increases of Aβ1-42 and Aβ1-40 peptides in N2a/APPswe and N2a/APPwt cells compared to controls. In line with the increased expression levels, the histone H3 in the promoter regions of the PS1 and BACE1 was found to be hyperacetylated in N2a/APPswe cells, which resulted in enhanced transcriptional activities. However, there was little change in H3 acetylation to the genes' promoters in N2a/APPwt cells compared to the controls. The results indicated that H3-acetylation-dependent-epigenetic regulation mediates PS1 and BACE1 transcriptional activation in familial AD but not in sporadic AD. Similar histone H3 hyperacetylation of the BACE1 promoter has been reported in APP/PS1/tau triple transgenic AD mice [9]. In addition, we found that the cellular histone acetylation is elevated in N2a/APPswe cells, and an increasing trend of H3 acetylation in N2a/APPwt cells, which is consistent with the observation that histones are hyperacetylated in human neuroblastoma SH-SY5Y cells by the Aβ peptide [11], [12]. We also found that the increasing trend of cellular histone H3 acetylation is consistent with the increased expression of Aβ1-42 and Aβ1-40 peptides in N2a/APPswe and N2a/APPwt cells. This data suggested that the cellular histone hyperacetylation may regulate the genes that produce the Aβ peptides, and the underlying complex mechanisms need to be further explored and tested.

It has been well established that histone acetyltransferase p300 evokes gene transcription by modifying histone acetylation or interacting with transcription factors such as NF-κB, p53, caspase, CREB, and PCAF [13], [24]–[26], contributing to multiple transcription events in neurodegenerative disorders. We have demonstrated here that p300 protein and HATs activity are increased in N2a/APPswe cells but not in N2a/APPwt cells compared to control cells. This is consistent with the previous report that mutation in another familial AD gene PS1 upregulates CBP/p300 protein in murine neuronal cells [7]. Saura *et al*. reported that PS1/2 knock-out mice display a decreased expression of CBP/p300 [8]. These studies suggested that the CBP/p300 overexpression may be associated with the familial AD pathology. Nonetheless, further investigation is needed to confirm whether APPCBP/p300 activation.

There are some conflicting results about the relationship between the CBP/p300 expression and AD development. Rouaux *et al*. pointed out that in the context of AD pathology APP signaling pathway activation is induced by an antibody directed against the APP extracellular domain, showing a critical CBP/p300 loss [13]. However, this is not the case in a familial AD model where it crarries APP or PS1/2 mutations. In addition, several studies suggested that CBP/p300 promotes the long-term memory signaling mechanism [14], [27]. Nontheless, no study has focused on the direct correlation of CBP/p300 function with memory deficits in the context of AD pathology.

We chose curcumin, a natural HAT-p300 specific inhibitor, to further examine whether HAT-p300 is associated with hyperacetylation of PS1 and BACE1. Our data demonstrated that the p300 expression is strongly attenuated when cells are treated with curcumin, and curcumin significantly suppresses the enhanced HATs activity in N2a/APPswe cells. Similar inhibitive effect of curcumin on p300 has also been found in heart diseases, cancer and other illnesses [16], [28], [29]. Our studies showed that p300-HAT inhibitor curcumin abrogates H3 hyperacetylation of PS1 and BACE1, along with attenuation of the high expression of these genes, supporting our hypothesized mechanism that p300 HAT enzymatic activity is recruited to the PS1 and BACE1 promoters, leading to transcription activation. The novel effect of curcumin on suppressing p300 expression may provide the rationale for a potential treatment regimen of AD using curcumin.

Several studies suggested that curcumin suppresses the production of Aβ *in vitro* and *in vivo* due to its anti-inflammatory and antioxidant effects [30], [31]. Zhang *et al*. reported that curcumin decreases PS1 activity by inhibiting GSK-3 to reduce Aβ production in human SH-SY5Y neuroblastoma cells transfected with APPswe [32]. These studies indicated that there might be other mechanisms underlying the effect of curcumin on suppressing the expression of PS1 and BACE1 and their downstream products Aβ1-42 and Aβ1-40 in N2a/APPswe cells.

**Figure 5:**
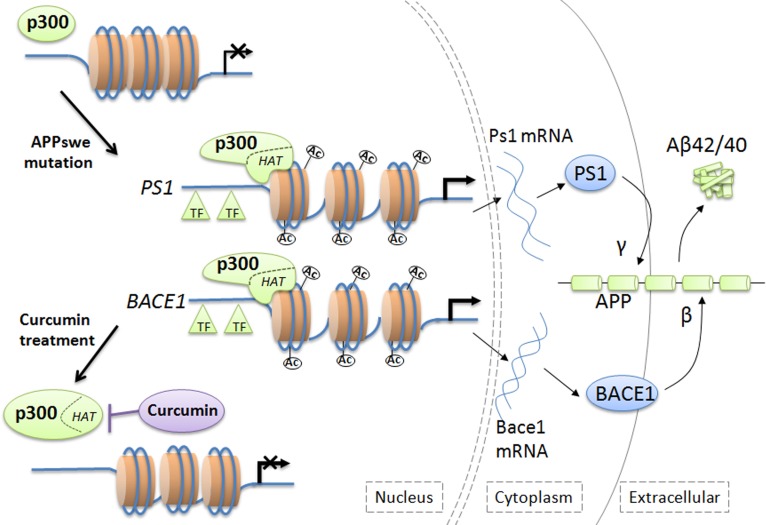
p300-dependent transcription activation of PS1 and BACE1. p300 HAT activity facilitates the histone acetylation of the PS1 and BACE1, accompanied by a more open chromatin structure that further facilitates accessibility by transcription factors to DNA-sequence of the genes, which in turn, leads to enhancement of the transcriptional activation. HAT: acetyltransferase activity; TF: transcription factor.

Besides targeting the histones of chromatin or indirectly recruiting transcription factors, p300 could directly interact with gene loci to stimulate transcription of specific genes [33]. Indeed, we demonstrated for the first time that p300 directly binds to the promoter regions of PS1 and BACE1 in N2a/APPswe cells but not in N2a/APPwt cells, and the binding is reversible by curcumin treatment in N2a/APPswe cells. It is possible, or even likely, that the APPswe mutation induces the p300 interaction with the genes and the HAT activity of p300 stimulates the PS1 and BACE1 promoter histone hyperacetylation, followed by an unwinding of the DNA -sequence that exposes the promoter regions and makes them accessible for other transcription factors to activate the transcription (Figure 5). Further studies, however, are needed to confirm such hypothesis on the DNA accessibility to the transcription factors within the gene promoters.

In summary, we have shown a novel mechanism of transcription activation of AD-related genes PS1 and BACE1 where p300 HAT-activity-dependent recruitment to the promoter regions occurs. Our studies provided a new insight for the histone modification in AD pathology, suggesting a potential therapeutic strategy targeting p300 function might be a useful treatment for familial AD patients.
